# Visualization of clinical teaching citations using social network analysis

**DOI:** 10.1186/s12909-021-02643-6

**Published:** 2021-06-16

**Authors:** Hakimeh Hazrati, Shoaleh Bigdeli, Seyed Kamran Soltani Arabshahi, Vahideh Zarea Gavgani, Nafiseh Vahed

**Affiliations:** 1 Center for Educational Research in Medical Sciences (CERMS), Department of Medical Education, School of Medicine, Iran University of Medical Sciences, Tehran, Iran; 2 Department of Medical Library and Information Sciences, School of Management and Medical Informatics, Tabriz University of Medical Sciences, Tabriz, Iran, Tabriz Health Services Management Research Center, Tabriz University of Medical Sciences, Tabriz, Iran; 3 Research Center for Evidence Based Medicine, School of Medicine, Tabriz University of Medical Sciences, Tabriz, Iran

**Keywords:** Hospital teaching, Round teaching, Centrality, Density, Social network analysis

## Abstract

**Background:**

Analyzing the previous research literature in the field of clinical teaching has potential to show the trend and future direction of this field. This study aimed to visualize the co-authorship networks and scientific map of research outputs of clinical teaching and medical education by Social Network Analysis (SNA).

**Methods:**

We Identified 1229 publications on clinical teaching through a systematic search strategy in the Scopus (Elsevier), Web of Science (Clarivate Analytics) and Medline (NCBI/NLM) through PubMed from the year 1980 to 2018.The Ravar PreMap, Netdraw, UCINet and VOSviewer software were used for data visualization and analysis.

**Results:**

Based on the findings of study the network of clinical teaching was weak in term of cohesion and the density in the co-authorship networks of authors (clustering coefficient (CC): 0.749, density: 0.0238) and collaboration of countries (CC: 0.655, density: 0.176). In regard to centrality measures; the most influential authors in the co-authorship network was Rosenbaum ME, from the USA (0.048). More, the USA, the UK, Canada, Australia and the Netherlands have central role in collaboration countries network and has the vertex co-authorship with other that participated in publishing articles in clinical teaching. Analysis of background and affiliation of authors showed that co-authorship between clinical researchers in medicine filed is weak. Nineteen subject clusters were identified in the clinical teaching research network, seven of which were related to the expected competencies of clinical teaching and three related to clinical teaching skills.

**Conclusions:**

In order to improve the cohesion of the authorship network of clinical teaching, it is essential to improve research collaboration and co-authorship between new researchers and those who have better closeness or geodisk path with others, especially those with the clinical background. To reach to a dense and powerful topology in the knowledge network of this field encouraging policies to be made for international and national collaboration between clinicians and clinical teaching specialists. In addition, humanitarian and clinical reasoning need to be considered in clinical teaching as of new direction in the field from thematic aspects.

## Background

It is important for research funding institutions and higher education organizations to identify the trend of an academic field of study, the influential contributors in the field and the future direction of the fields. Medical science is a rapidly evolving field and a foremost pragmatic science that uses scientific findings and research outputs. Clinical teaching is one of the most important components of medical education [1]. Because, education of medical science based on theory cannot overcome the problems of healthcare system and health care professions. Clinical training requires evidence –based treatment methods and practices. Clinical teaching is influenced by different components such as characteristics and roles of the medical teacher, clinical teaching behaviors, teaching methods in the clinical setting, and the roles of the clinical environment educational system [2].

To reach an evidence- based practice in the field of clinical teaching, all of the components of the field have to be covered with robust researches. Clinical practitioners and clinical teaching experts need to collaborate in team working, teaching and conducting researches to improve the medical knowledge transformation into young generation. It is important to explore team-working, and to identify the influential researchers, organizations in the field for future collaboration and research development as well as the current focus of researches and future direction. Therefore, it is important to study and analyze the scientific map of clinical teaching research outputs to understand the relationships, dominant subjects and key researchers in this field.

Analysis of research results in the field of clinical teaching helps clinical instructors improve their clinical teaching skills and perceive what has been considered in the past in order to recognize what could be investigated further in the future [3].

In addition to the usual statistical techniques of data analysis, recent increases in computational power have increased the accessibility of social network analysis methods for application to medical education research [4]. The Social Network Analyses (SNA) are used for investigation of the relationships between all components of a field as nodes and links in a network model. Therefore, SNA is a mean used to identify the knowledge systems of each field by analyzing and visualizing the previous research results through the literature in the field [5]. It helps to visualize and analyze the structural patterns of the social relations and presents co-authorship networks and scientific map to draw a roadmap for the future research and practice [6]. According to the social network theory, dissemination of opinions and information critically depends on the persons who have the highest relation within a group, and known as role players. Core components of medical education in micro level (clinical practitioners or clinical teachers) or macro level (curriculum developers, health policymakers) and each research contribution (i.e. paper) are known as the nodes and links of the social network of medical education. Efficiency of the information flow within the network is recognized by the density of the network, or the points that are connected within the network [7]. Review of the previous researches showed a perceived gap in identifying the density in the co-authorship network and the thematic network of clinical teaching as an independent field of study. Analyzing the core and growing subjects in publications of a single sources like the Korean Journal of Medical Education, by SNA approach [8] or single method of teaching like problem based learning (PBL) in medical education by scientometrics methods [9] are comminuted researches that cannot give a complete image of relationships in clinical teaching. Ji YA (2015) has investigated the trend of medical education research articles published in PubMed by SNA to understand the interaction of medical education subjects in the framework of complex systems theory [5]. To date, there have been no independent and up-to-date study of social relations and behaviors of components of clinical teaching based on comprehensive research out- puts in clinical teaching field using SNA.

Knowledge is expanding faster than our ability to assimilate and apply it effectively; and this is as true in education and patient care as it is in research [10]. In 2017, it was said that, the half-life of medical knowledge is about 18–24 months, and it is projected that in about 4 years that half-life will be only 73 days [10]. It was estimated that the doubling time of medical knowledge in 2020 it is projected to be 0.2 years—just 73 days. Considering the rapid changes in medical knowledge and the half-life of medical researches, it is essential to analyze the newer researches in the field from the broader sources to be able to visualize a possibly up-to-date and complete knowledge image from the co-authors and subject focus in clinical teaching.

Therefore, this study aimed to investigate the research outputs of clinical teaching and visualize the collaboration networks of authors, countries and the subject clusters in the field of clinical teaching.

## Methods

### Study design

In this study, we used Social Network Analysis (SNA) to analyze and visualize collaboration networks and subject clusters of the field of clinical teaching. Both the macro and micro level indicators have been used in this visualization.

The macro level indicators describes overall characteristics of the networks and examine the topology and the possible performance of the social structure [11]. We measured ‘density’ and ‘clustering coefficient’ (CC) of the networks for analyzing the network at macro level. Network density defines as the ratio of possible ties which are recognized among the participants of network and it measures the degree consistency of the nodes. The density is scored between zero and one, being closer to one indicates that the network is more coherent and the relations between nodes are denser [12–14]. The Clustering coefficient is defined as the ratio of the numbers of links around a node and possible links in the network. This index also is scored between zero and one. The numbers close to one shows a higher rate of connections with and within the colleagues. We also used ‘centralities” at micro level metrics to identify the performance of each node (person or organization) in the network [11]. Centrality measures connections among nodes in the network and describes structure of a network. We used three indicators of centrality in this study including Degree, Closeness, and Betweenness centralities [15].

#### Degree

This indicator shows the number of ties that each node has made in the network with other nodes. It indicates that how many direct connections each node has to other nodes in the network. Authors/countries with a high degree centrality can be considered to be active members and have a higher number of corporations with other nodes in the network. Indeed a network member with a higher degree is a popular actor of network and has more ability to influence others [14, 16, 17].

#### Closeness

This indicator emphasizes the structural position of nodes in the network and is defined as total geodesic distances to all other nodes [16]. Accordingly, authors with high closeness centrality are actors of the network, that receive information quickly and provide its distribution and accessibility throughout the network [13, 14].

#### Betweenness

This indicator is based on the number of the shortest paths that cross through a node. Authors with high betweenness are the most influential persons in the network and have most connections to different groups [13, 16].

### Search strategy and study selection

We searched Web of Science (maintained by Clarivate Analytics), Medline (produced by the U.S. National Library of Medicine) through PubMed, and Scopus (provided by Elsevier) (Diagram1). We also searched the main journals in clinical teaching manually, such as: Medical Teacher, Medical Education, BMC medical education, Academic Medicine, Clinical Teacher, and Teaching and Learning in Medicine. Picking and aggregating valid words in literature-based studies like systematic reviews, scientometric studies and social network analysis is essential and key parts of study. Selection of inappropriate keywords may lead to collection of heterogeneous data [18]. Therefore, we used consensus of experts to select and validate the eligible key words in the search strategy. The expert panel consisted of faculty members of medical education, clinical librarianship and a medical education PhD candidate. Once the keywords were identified they were combined based on a search strategy designed by a clinical librarian and the search was performed consequently. The main query terms were “Hospitals teaching” “Rounds teaching”, “Students, Medical”, “education, Medical”, “medical teacher”,” clinical teaching behavior”, “clinical environment “and “clinical teaching “based on Medical Subject Headings (MeSH), developed by the U.S. National Library of Medicine [19] and natural language. We performed the search in April 16th, 2018. We limited the search results to the fields of title, abstract, and keywords. We limited the date to articles published from “1980/01/01 to “2018/04/31. Since the year 1980, the second wave of reforms in universal medical education was occurred based on the Flexner’s suggestions. The reforms has led the scientists to comprehend the difference between andragogy and pedagogy learning and considered concepts such as student-centered, integration in medical education, problem based learning, and community-oriented medical education [20].

We used “PICo”(Population, phenomenon of Interest, Context) for formulating research questions.

#### Population

All publications on the clinical teaching in the field of medicine.

#### Concept

Characteristics and roles of the medical teacher, clinical teaching behaviors, teaching methods in the clinical setting, and the roles of the clinical environment.

#### Context

Clinical teaching can be applied in every discipline, whether in internal medicine, surgery, emergency, obstetrics, or in any other specialty department in teaching hospitals around the world. Clinical teaching can take place in all sites where patients are exposed to medical care, including inpatient wards, outpatient care settings, community centers [21] Therefore, we did not differentiated the variety in context and all of what made the context of study.

### Inclusion and exclusion criteria

We included the studies that provide data on clinical teaching, and those on the clinical assessment are excluded. The clinical teaching can be applied in the special hospital in every discipline such as: internal medicine, pediatric, emergency, and psychology or in every other specialty departments or in primary health centers and rural health centers, all settings have been considered eligible to be included in this study. More, the studies on dentistry education and paramedical clinical settings, literature reviews, letter to editors, conference abstracts, dissertations, and nonprofit organization reports were excluded because they did not encompass original data. We also excluded the authors with the less than two articles, based on the thresholds used in SNA studies, to visualize the co-authorship network and representation of the a suitable network [13].

**Fig. 1:**
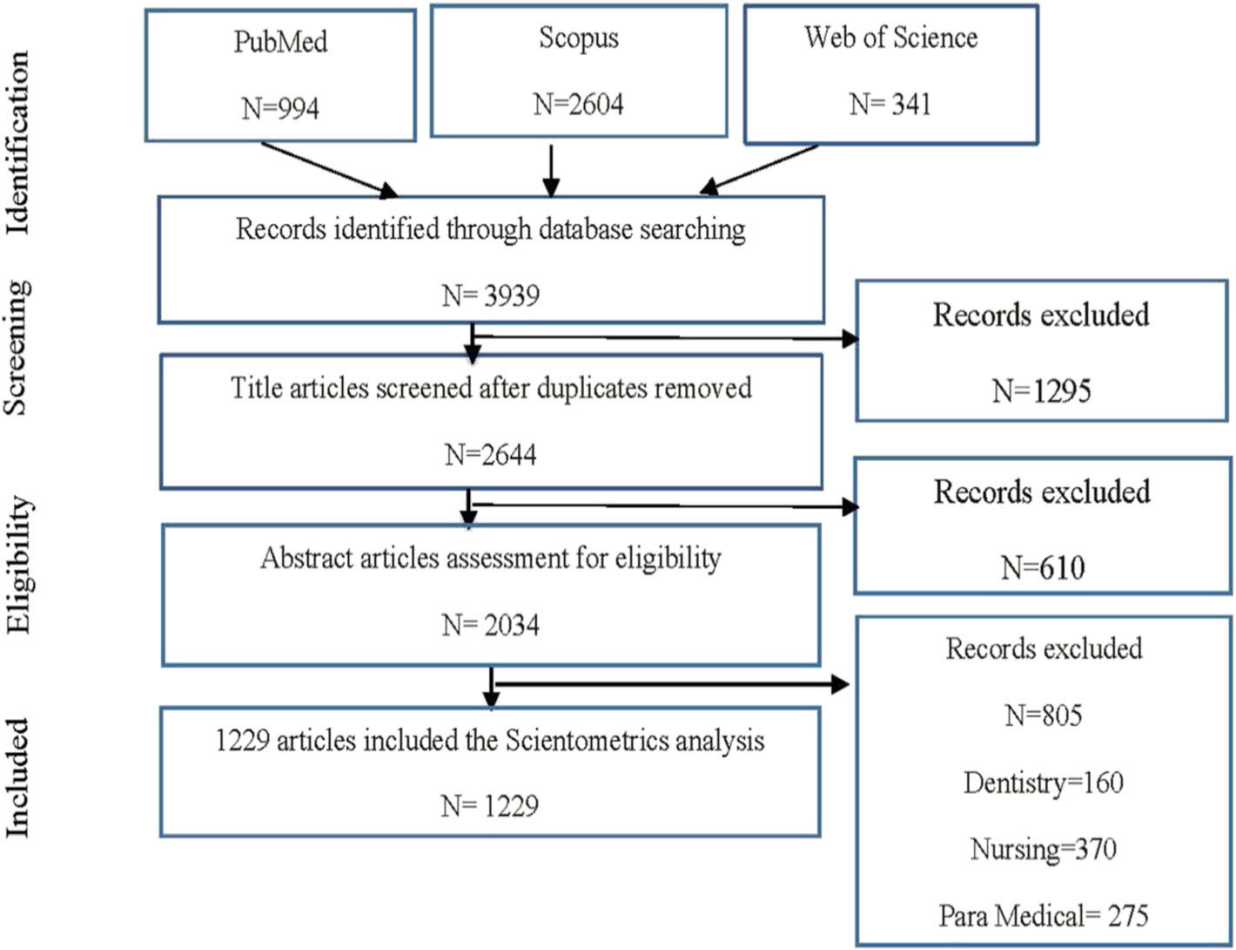
PRISMA flowchart of articles selection

The search strategy identified 3939 articles. After removal of duplicates in the databases and the assessment of title and abstracts, 1229 articles were included into the scientometrics analysis (Fig. 1).

#### Organization of the data

Retrieved data from selected databases imported to Excel 13 after sorting data by titles according to the alphabetic order. We used Levenshtein Distance technique to remove duplicated papers collated from different databases, publication year, volume and number of similar titles by similarity > 75% [22]. In this stage, two researchers independently reviewed title and abstracts of the retrieved articles.

#### Standardization of names

One of our major concerns in the refining process was the variations appeared in the name of authors and countries. In SNA, the variations of names and nomenclatures lead to redundancy in nodes and clusters. In addition we used the data preparation and cleansing method introduced in previous SNA literatures [14]. We considered the whole list and distinct words that needed modification. Then, for standardization of literature retrieved from Web of Science, we used the author names appearing in the field of Author Address - C1. We found name variety for some of the authors. In order to correct the name variety for authors we checked the spelling of the author name from ORCID ID for SCOPUS based articles. For articles retrieved from PubMed/Medline, we searched the name with same affiliation from the Google. We also unified different format of country names such as, the USA and the United States of America and the US by accepted formats in the research team. Standardization of topics performed by the use of MeSH terms (Medical Subject Headings) and selected rational topic for specific topics have not equal MeSH terms Also, we deleted numbers or symbols such as %, #,&, >,” and merged single and plural forms of keywords, converted abbreviations to full form by checking them with a list of synonyms [14]. Two of the researchers independently revised the list of names, nomenclatures and words. To prepare the adjacency matrixes, the names of authors, countries, and Keywords were separately imported to the Ravar PreMap software.^1^ The cleansing and standardization were applied based on the mentioned strategies in the software. We also reviewed the background education and affiliation of authors to identify whether the authors of clinical teaching articles are from the clinical background or the educational background.

#### Visualizing the data

##### Visualizing the collaboration network of authors and countries

Co-authorship networks revealed relationships among authors. In this network, nodes are indicator of authors and the links between two nodes identifies co-authorship between the two authors. By using co-authorship network the degree of relationships and the influence of different scientists or groups within the special research filed is described [13].

Ravar PreMap software was used to prepare bibliometric data and creating co-occurrence matrix of keywords that could be read by the network analysis software. The UCINET software version 6.421 was used to convert the data to a format which could be read with Netdraw to visualize the collaboration network of authors and countries.

##### *Visualizing the* subject clusters

Cluster analysis was done to keywords to be revealed in the clinical teaching network and the primary cluster. A cluster is a set of closely related nodes with each node in a network assigned to one cluster (Waltman, Van Eck, & Noyons, 2010, p.7) Co-word analysis is based on the principal that if two keywords situate concurrently in a paper, it means that there is a closer relationship between them [23]. To recognize the main concepts in clinical teaching we used co-word analysis and visualization of keywords using VoSViewer.^2^

## Results

**Fig. 2:**
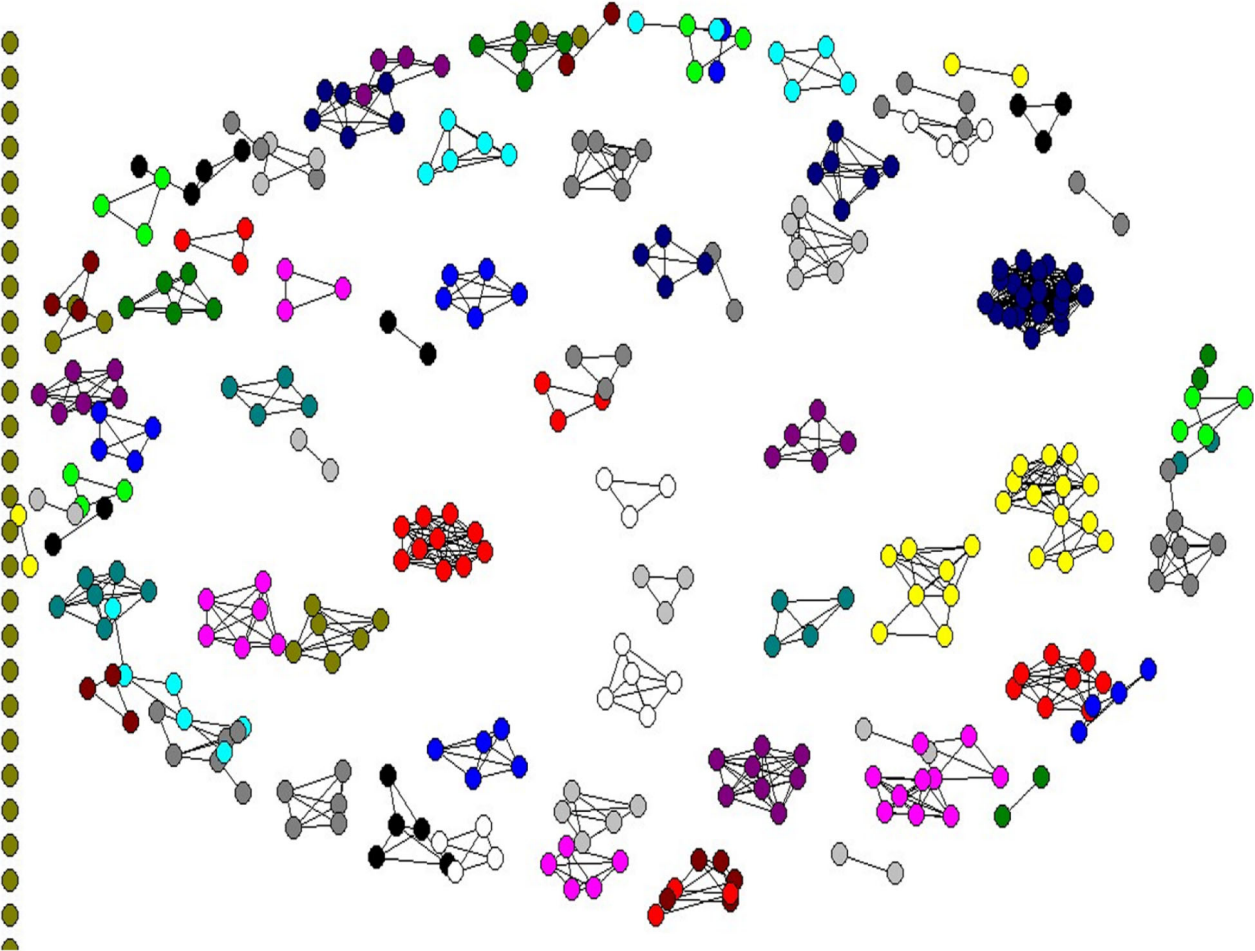
The co-authorship network of authors in clinical teaching research

Findings of the study show that a total number of 2767 authors contributed in writing 858 articles in clinical teaching. Co-authorship network consists of 367 authors (nodes) and 1380 co-authorships (link). As seen in Fig. 2, there are many tiny and independent networks in this system. The two largest networks consisted of 20 authors and 380 co-authorship, and the next biggest network consisted of 13 nodes and 92 co-authorship [Fig. 2].

The micro level analysis, the clustering coefficient of the co-authorship network was 0.749 and the density of network was 0.0238.

**Fig. 3:**
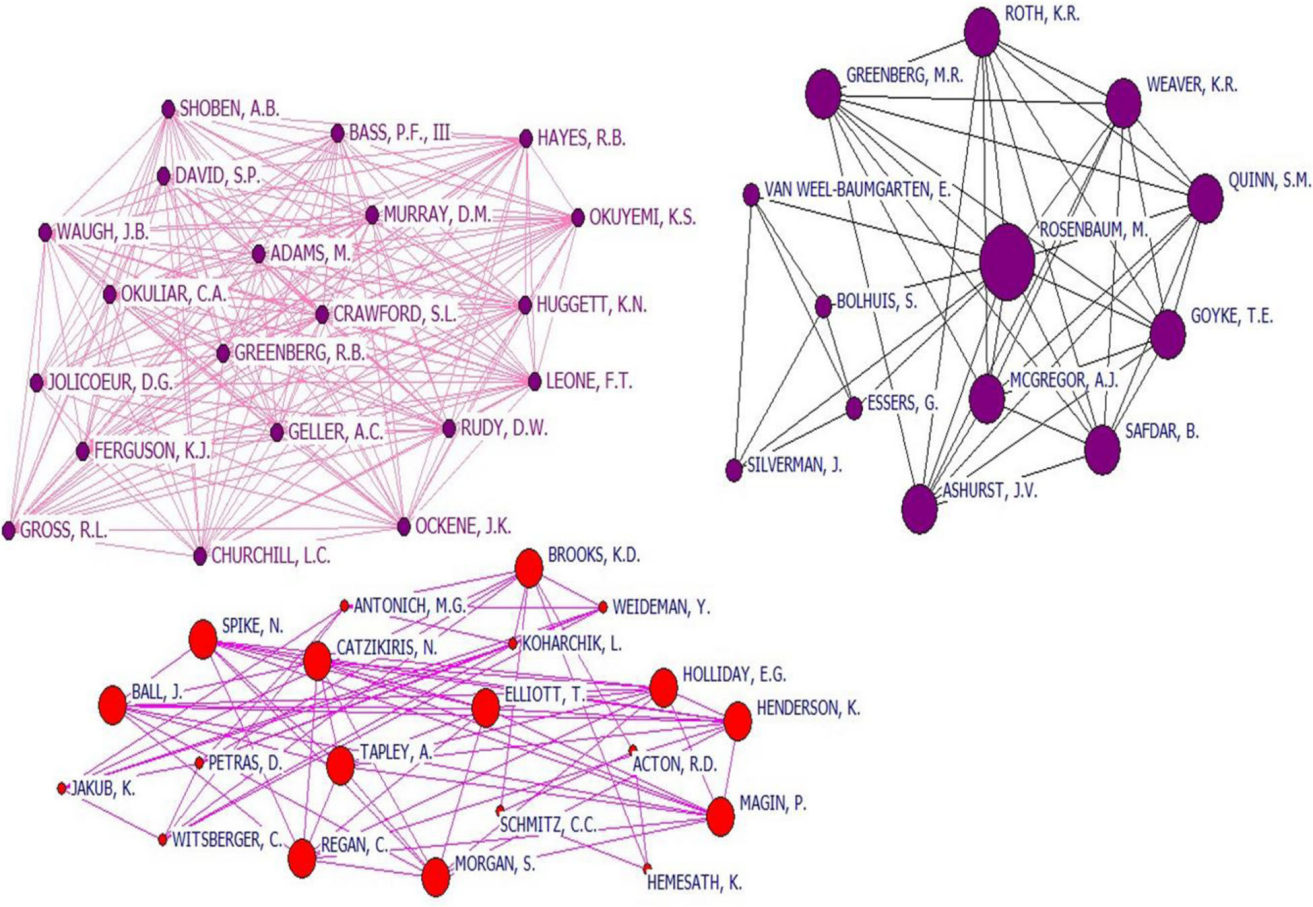
The three largest co-authorship network of authors in clinical teaching research

The micro level analysis, the network consists of 13 nodes constructed of two networks. One of them consists of 4 nodes (authors of medical education researches) and the other has 9 nodes (researcher of emergency specialists). Rosenbaum with the greater betweenness, closeness, and degree centrality is known as the most important node in this study and connects the two networks of authors in clinical teaching. Rosenbaum has also the highest frequent connection with other authors in this network [Fig. 3].

Network consists of 20 nodes, specialty authors were from Public health (*n* = 6), Family and Community Health (*n* = 4), Internal Medicine & Pediatrics (*n =* 4), lung disease sub-specialty (*n* = 3), Biostatistic (*n* = 2), and Medical education researcher (*n* = 1). All scientists of this network were from the USA. The second network consists of 20 nodes, specialty authors were general practitioners (*n* = 11), Nurses (*n* = 5), and surgery (*n=*: 4). Researchers of this network were from Australia and the USA [Fig. 3].

Centrality of the nodes was examined by using: degree, closeness, and betweenness indicators; 20 authors have the same degree and closeness. Adams, M., Churchill, L.C.,Crawford, S.L.,David, S.P.,Geller, A.C., Hayes, R.B., Jolicoeur, D.G., Leone, F.T., Ockene, J.K., Okuyemi, K.S., Bass, P.F., III, Greenberg, R.B., Gross, R.L., Huggett, K. N, Murray, D.M., Okuliar, C.A., Rudy, D. W, Shoben, A. B, Waugh, J.B., Ferguson, K.J. with 5.177 degree and 0.287 closeness are the leading and active members in the clinical teaching fields. In regard to normalized betweenness centrality, the most influential author in the network was Rosenbaum, M (0.048). He is Associate Professor affiliated to the Office of Consultation and Research in Medical Education of Iowa Carver College of Medicine USA. Table 1 show the most important authors based on centrality measures.

**Table 1:** Top 10 Authors in centrality and productivity in the clinical teaching

N	Author	Betweenness	Degree	Closeness
1	Rosenbaum, M.	0.048	3.270	0.281
2	Brooks, K.D	0.027	2.452	0.279
3	Teunissen, P.W.	0.010	1.907	0.277
4	Papp, K.K.	0.009	1.090	0.275
5	Walters, L.	0.009	1.090	0.275
6	Anderson, C.R.	0.007	1.635	0.276
7	Campbell, D.	0.006	0.817	0.275
8	Elnicki, D.M.	0.006	0.817	0.275
9	Scherpbier, A.J.J.A.	0.003	1.635	0.277
10	Hill, A.E.	0.003	0.817	0.274
11	Kennedy, M.	0.003	0.817	0.274
12	Wang, W.	0.003	0.545	0.274
13	Zhao, X.-L.	0.003	0.545	0.274

### Visualizing the collaboration network of countries

**Fig. 4:**
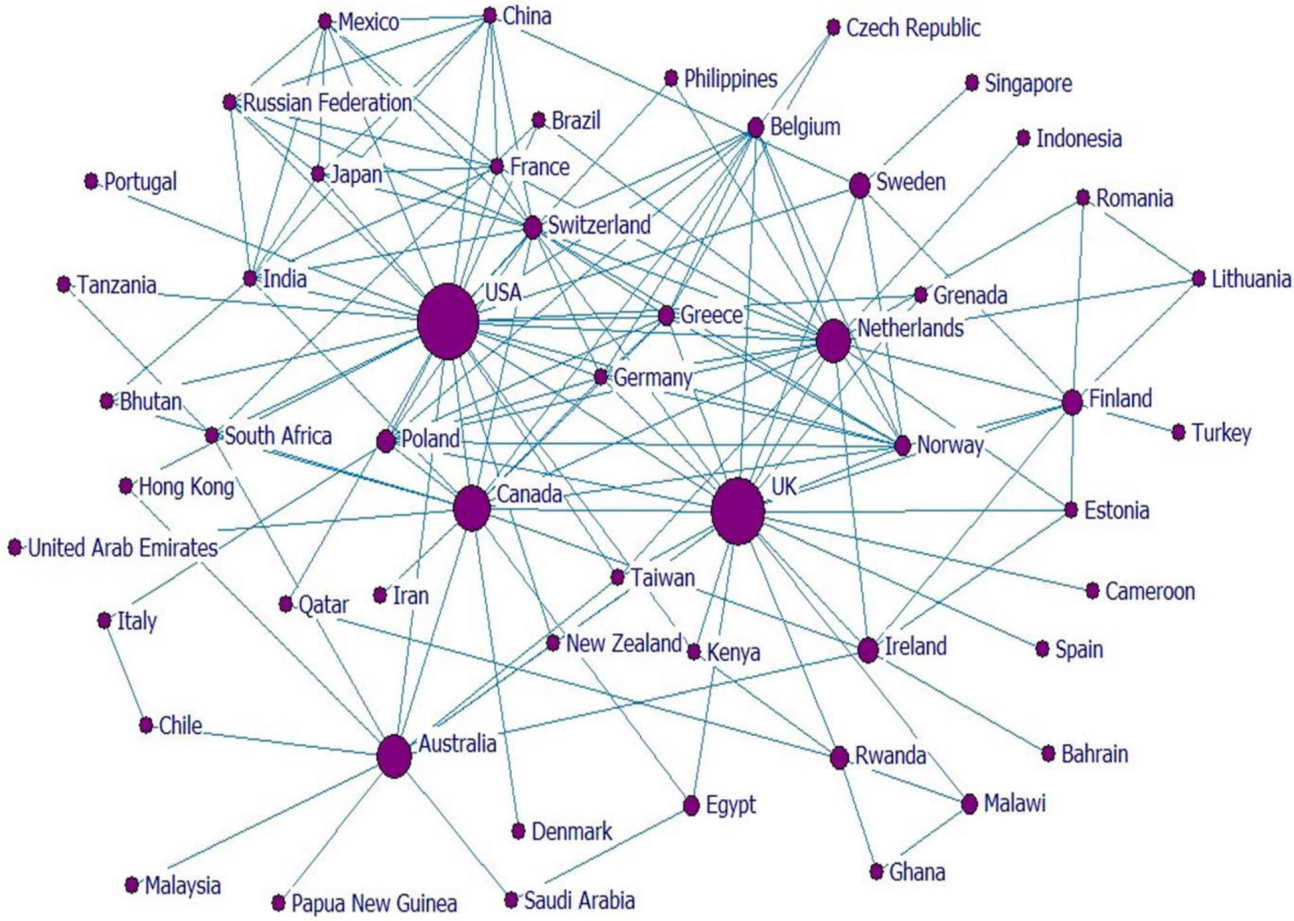
The co-authorship network of countries in clinical teaching research

In this collaboration network of countries (the nodes are country names and the lines linking pairs of them together) showed the authors who affiliated to at least one article. The size of a node was comparative to its normalized betweenness centrality. The international co-authorship of the countries participated in publishing articles in clinical teaching consisted of 54 countries and 308 co-authorship (links ([Fig. 4].

In the micro level analysis, the density of the collaboration network of countries was 0.176, which shows that the general consistency of network is low and only 17.6% of all possible relations have been presented. The network clustering coefficient was 0.655; it means that if two authors of the countries A and B have co-authorship with the authors of country C separately, there is about 65.5% probability that authors of countries A and B to have a co-authorship in the near future.

The top 10 countries contributed in producing papers in clinical teaching based on centrality measures has been shown in (Table 2). The USA marks the highest stand based on normalized centrality indicators [betweenness 32.223, closeness 66.250, and degree 52.475]. It has a central role in collaboration network of countries and has the vertex co-authorship with other countries in clinical teaching.

**Table 2:** Top 10 countries in centrality and productivity in the clinical teaching filed

N	Country	Betweenness	Degree	Closeness
1	USA	32.223	54.717	66.250
2	UK	27.704	43.396	63.855
3	Canada	16.720	33.962	58.242
4	Australia	15.120	22.642	52.475
5	Netherlands	15.034	35.849	56.989
6	Finland	5.598	16.981	45.299
7	Ireland	4.985	13.208	48.182
8	Sweden	4.480	11.321	47.321
9	Switzerland	3.822	28.302	53.535
10	Poland	3.423	18.868	51.456

The UK (27.704) illustrated as the second country with top position in the clinical teaching collaboration network based on centrality indicators [betweenness 27.704, closeness 63.855, and normalized degree centrality 54.717], and Canada was in the third rank [betweennness 16.720, closeness 58.242, and degree centrality 33.962]. Australia and the Netherlands were ranked fourth.

It was also found that the USA has the nearest geodesic path between other countries that marks it as an active country, closer to the other nodes, with an influential role and high collaborations with other countries.

### Identifying and visualizing subject clusters

**Fig. 5:**
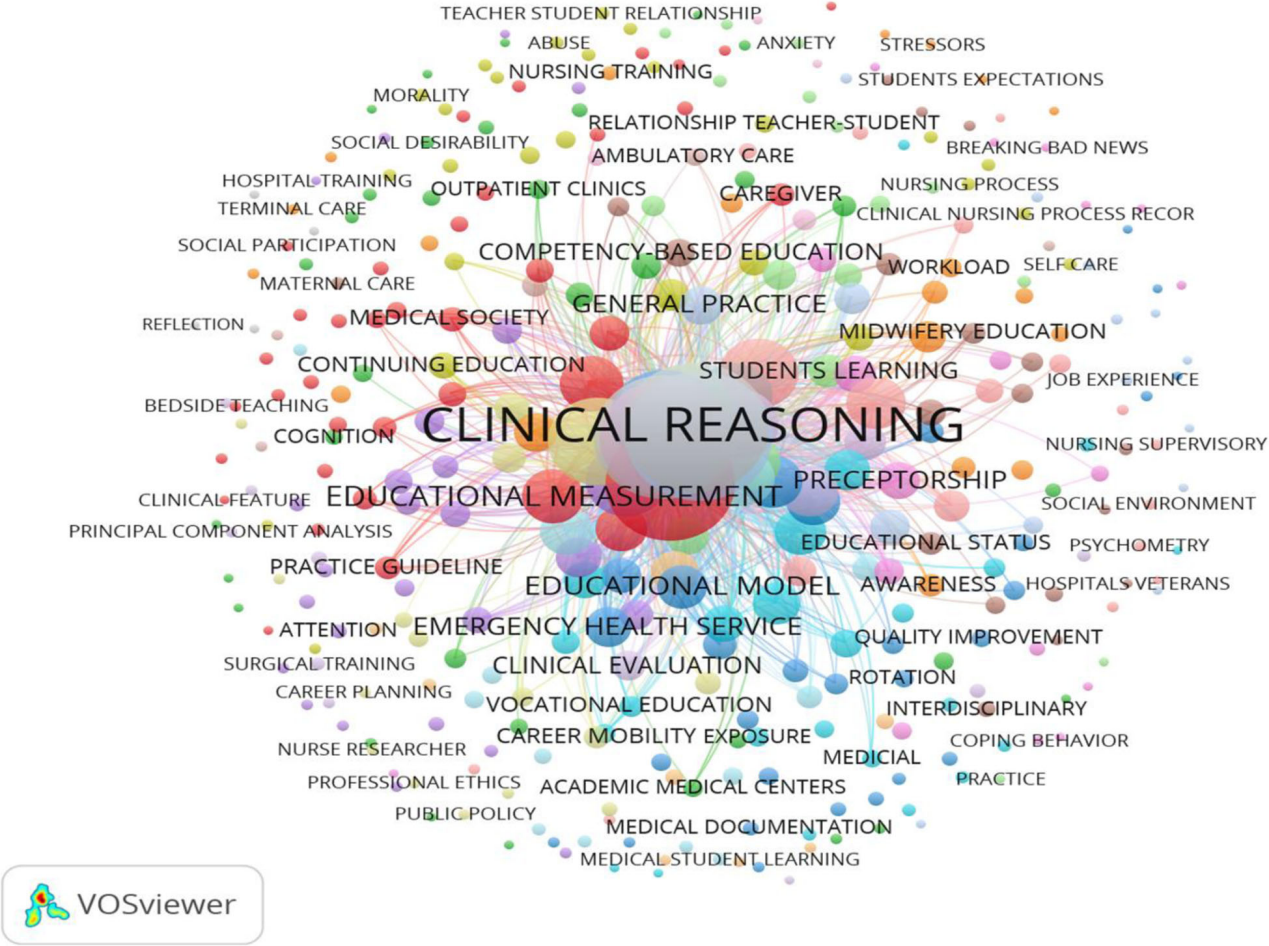
Co-occurrence network of keywords in clinical teaching research

A total number of 1150 keywords constructed the thematic network of clinical teaching research outputs. 356 keywords with 6741 link were identified based on word co-occurrences ≥2 that nested in 19 subject clusters. The visualization of keywords drawn by VOSviewer is shown in (Fig. 5).

The 20 high frequently occurred keywords based on centrality indicators have been showed in (Table 3).

**Table 3:** The 20 high frequently occurred keywords based on centrality indicators

N	Keywords	Frequency	Betweenness	Degree	Closeness
1	Humanism	137	13.407	97.753	97.802
2	Clinical reasoning	139	13.213	95.506	95.699
3	Clinical education	129	11.208	92.697	93.194
4	Cooperative behavior	102	7.980	83.708	85.990
5	Medical student education	105	7.716	82.865	85.372
6	Tutorial strategies	72	4.411	69.101	76.395
7	Medical education	59	3.287	61.236	72.065
8	Clinical clerkship	62	3.125	60.674	71.774
9	Learning and teaching	50	2.174	52.247	67.681
10	Clinical competence	49	2.145	54.213	68.593
11	Adult education	36	1.852	49.719	66.542
12	Curriculum development	38	1.788	49.157	66.294
13	Psychiatry education	38	1.701	46.067	64.964
14	Communication skill	32	1.661	47.753	65.683
15	Professionalism	21	0.904	38.764	62.021
16	Attitude to health	23	0.808	37.360	61.485
17	Procedures	24	0.559	32.303	59.631
18	Nursing education	22	0.509	28.933	58.456
19	Education measurement	20	0.448	31.461	59.333
20	Human experiment	20	0.254	26.404	57.605

The 19 subject clusters recognized as sub-networks in the clinical teaching is presented in Table 4. The first and the biggest cluster contains 16 phrase, in which the term “Humanism”, with 137 frequency and 347 link was the most frequently co-occurred terms (Table 4), The second bigger cluster contained 4 phrase including Clinical education teaching, Clinical reasoning, Reflection, Reflective practice development, in which the phrase “Clinical reasoning”, with frequency of 139 and 348 links, “Clinical education” with frequency of 129 were the most frequently co-occurring key terms in this cluster (Table 4).

**Table 4:** Subject Clusters in clinical teaching

No.	Name of the Cluster	Number of items, links and Frequents	Keywords within the cluster
1	Clinical reasoning	4 Items, 348 link, 3770 total link, 139 Frequents	Clinical education teaching, Clinical reasoning, Reflection, Reflective practice development
2	Humanism	(16 items) (link = 347 total = 3764) Frequents: 137	Decentralized clinical training, Humanism, General practice, Job experience, Learning curve, Medical expert, National health insurance, National health service, Organization and management, Personal value, Primary health care, Public health service, Secondary health care, Specialist outreach, Tertiary care center, workshop
3	Cooperative Behavior	38 Items, 297 link, 2904 total link, 102Frequents	Adolescent psychiatry, Attitude to computer, Bedside teaching, bedside learning, Blended Learning, Capacity building, Caregiver, clinical future, Computer- assisted instruction, Conceptual framework, Cooperative behavior, Delivery of health care, Educational measurement, Health care personal, Health program, Health promotion, Integrated health care system, Learning theory, Medical information system, Medical society, Medical student core curriculum, Needs assessment, Online Curriculum, Online system, Patient referral, Practice guide line, Procedures, Referral and consultation, Resident as a teacher, Self-evaluation, Shared learning, Social media, Social participation, Society medical, Standardization, Teacher training, teaching assessment, Trainee- as – teacher, Treatment planning
4	Medical student education	18 Items, 170 link, 1145 total link, 105 Frequents	Adaptation psychological, Breaking bad news, Clinical teaching methods, Coping behavior, Core content, Emotional intelligence, Experiential learning, Hospital wards, Interview, Learning history, Medical student education, One minute preceptor, Oral communication, Pre- Residency, Problem based learning, team-based practice, Truth disclosure, Under graduate educ
5	Tutorial strategies	30 Items, 245 link, 2146 total link, 72 Frequents	Clinical examination, Clinical Observation, Continuous quality improvement, Crowding, Educational model, Emergency health service, Emergence ward, Expanding clinical capacity, Formative feedback, Hospital emergency, Knowledge, Medical care, Medical documentation, Medical history taking, negative feedback, Pattern recognition, Personal satisfaction, Physical examination, Quality control, Quality improvement, Recognition, Reinforcement, Rotation, Skill, Standard, Teaching management, Total quality management, Tutorial strategies, Two mint observation,
6	Medical education	16 Items, 217 link, 1726 total link, 59 Frequents	Career, Career mobility, Career planning, Course content, Health care, Health science, High fidelity simulation, Medical education, Mentoring, Nurse researcher, Patient safety administration, Professional ethics, Public policy, Sex characteristics, Staff development, Teaching materials
7	*Learning and teaching*	18 Items, 185 link, 1452 total link, 50 Frequents	Adolescent, Anxiety, Clinical learning, Clinical teaching environment, Documentation, Early clinical experience, Electronic Portfolio, Field work, Information processing, Learning and teaching, Medical informatics, Practice, Professionalism, Responsibility, Socio economic, Students learning, Under graduate student, Virtual patients
8	Clinical competency	34 Items, 158 link, 2234 total link, 49 Frequents	Academic achievement, Academic advancement, Academic medical center, Brain and education, Clinical competency assessment, Clinical educator, Cognition, Continuing skills, Disease severity, Educational environment, Experience, Faculty development, Faculty practice, follow up, Health belief, Hospital care, Job satisfaction, longitudinal integrated clerks, Medical specialist, Normal human, Outpatient clinics, Patient comfort, patient satisfaction, Patient rooms, Peer mentoring, Personal experience, Postgraduate education, Promotion, Retention competence, social desirability, Teaching approaches, Teaching Competence, Tele medicine, University hospital
9	Adult education	15Items,176 link, 1142total link, 36Frequents	Adult education, Behavior, clinical clerkship, Clinical competence, clinical evaluation, Cognitive pre-training, Human experience, Individualization, Internal consistency, Intraoperative learning, Learning environment, Multimedia, Performance, Principal component, Surgical training
10	Curriculum development	26 Items, 174 link, 1172 total link, 38 Frequents	Attention, Audiovisual equipment, Behavior change, Behavior therapy, Clinical supervision, Computer simulation, constructive feedback, Cost control, Crossover procedure, Curriculum development, Cybernetics, Educational technology, Evidence-based medicine, Health care quality, Hospital training, Image quality, Learning system, Outcome assessment, Post graduate student, Simulation in clinical education, Skill retention, Student attitude, Student satisfaction, Teaching round, Virtual reality, Visual impairment
11	Psychiatry education	18 Items, 163 link, 1130 total link, 38 Frequents	Academic performance, Clinical learning environment, Clinical placement, Clinical remediation, Clinical teaching, Competency assessment, Educational pedagogy, Medical personnel, Nurse education, Nursing education research, Nursing supervisor, Pedagogical atmosphere, Personal Growth, Psychiatry education, Psychometric, Social environment, Socialization, supervisory relationship
12	Communication skills	25 Items, 169 link, 1040 total link, 32 Frequents	Career choice, Communication skills, Cultural diversity, Education professional, Exposure, Group dynamic, Health care planning, Health occupation, Health personal attitude, Health plan implementation, Interdisciplinary communication, Interdisciplinary research, Inter-professional clinical education, Inter-professional evaluation, Leadership, Medical profession, Medical, Organization and management, Patient care team, Perception, Program development, Rural health care, Stigma, Vocational education, Work based learning
13	Attitude to health	13 Items, 347 link, 3764 total link, 85 Frequents	Attitude to health, Continuity of patient care, Doctor patient relation, Health knowledge, attitude practice, Long term care, Longitudinal community based care, Medical student learning, Models psychological, Patient care, Physician patient relation, Verbal communication
14	Education measurement	5Items,58 link,125 total link,20 Frequents	Ambulatory care, Emergence contraception, Learning response, Mini-CEX, Peer group
15	Educational program	8 Items, 93 link, 428 total link, 12 Frequents	Clinical curriculum, Diagnostic imaging, Educational program, Medical imaging education, Medical radiology training, Objective structured clinical examination. Workflow 0.0806
16	Internship and resident	20 Items, 74 link, 336 total link, 12 Frequents	Awareness, Competency, Expectation, Health practitioner, Internship and resident, Mental stress, Nurse practitioner, Nursing staff, Nursing standard, Nursing Training, Patient attitude, Patient education, Quality of health care, Registered nurse, Stress psychology, Stressors, Student expectation, Support, Terminal care, Workload,
17	Human relations	26 Items, 72 link, 1246 total link, 72 Frequents	Abuse, Clinical nursing process record, Conflict, Continuing education, Ethics moral, Evaluation, Human relation, Medical assessment, Medical error, Medical Ethics, Medical Record system, Medical technology, Morality, Nurse patient relation, Nursing care, Nursing process, Professional misconduct, Professional student relation, Relationship teacher- student, Role Modeling, Scientific knowledge, Self-care, Self-directed learning, Social discrimination, Teachers student relation, Technology,
18	Competency-based education	19 Items, 49 link, 184 total link, 6 Frequents	Community health service, Community oriented education, Competency- based education, Educational status, Health center, Health service, Home care service, Hospital training, Hospital veterans, Interdisciplinary, Maternal care, Medical practice, Mental health service, Midwifery education, non- technical skill, Nursing home, Program evaluation, Specialization, Teaching skills
19	Motivation	4 Items, 31 link, 102 total link, 5 Frequents	Academic faculty, Clinical medicine, Goals, Motivation

The Third largest cluster contained 38 key-terms, in which phrase “Cooperative Behavior”, with the frequency of 102 and 227 links were the most frequently co-occurring phrase in this cluster (Table 4).

The fourth-largest cluster contained 18 key-terms, in which the phrase” Medical student education”, with frequency of 105 and 163 links was the most frequently co-occurring term in this cluster (Table 4).

The fifth-largest cluster contains 30 phrases, in which phrase” Tutorial strategies”, with a frequency of 72 and 245 link was the most frequently co-occurring term in this cluster (Table 4).

## Discussion

The collaboration networks of authors, countries and the subject clusters in clinical teaching field were visualized using SNA. Analyzing the findings revealed a perceived willingness for collaboration among authors in clinical teaching, based on the clustering coefficient of the co-authorship network. But the network was not cohesive and the density was low, so that about 23% of the total potential links in the network has been presented [Fig. 2]. This means that flow of information thought the network is not efficient and needs some intervention from the policy makers in clinical teaching research supports. This is in agreement with the results of studies performed in medical science network in which dense of network was (0.0806) and indicator of a low density [11].

One of the methods used to understand networks and their participants is to evaluate the location of actors in the network [24]. Our study showed that in clinical teaching co-authorship network Rosenbaum, is the cut point or connector of two peer networks of medical education researchers and physicians. Borgatti says the A cut-point is the node whose deletion increases the number of components by splitting the subgraph into two or more separate subsets [25]. In this network Rosenbaum M., presented the highest degree centrality, closeness centrality, and betweenness centrality among other clinical teaching researchers. This means that Rosenbaum has an influential position in the formation and cohesion of network and has connected different groups to each other, has performed a role of main actor in the network who quickly receives information and distributes it among others. Our data curation showed that she is affiliated to the Office of Consultation and Research in Medical Education. However, clinical teaching is key in transferring medical knowledge [1], but lack of time does not allow clinicians to conduct research in clinical teaching, but they can collaborate at least in researches to transfer their pure knowledge into practice. A Canadian research project revealed that close collaboration between clinical teachers (PhD students of medical education) and physicians improves the medical education researches and supports the medical education [20].

Our study is indicator of a low network clustering coefficient in the country level as well, so that only 17.6% of all possible relations have been presented [Fig. 4]. It means that if two authors of country A and country B have co-authorship with the author of country C separately, about 65.5% is possible that the authors of countries A and B will have a co-authored work in the near future.

The USA, the UK, Canada, Australia and the Netherlands had the highest co-authorship with other countries and were the most productive countries. Azer (2019) also identified that the leader universities in medicine have located in same countries [26]. We argue that based on the educational and scientific background of the US, the UK, and the Netherlands in pioneering medical education research centers [16] the result of our visualization is rational. Previous SNA studies in medical education also revealed that the United States, the UK, Australia and the Netherlands are the leading countries in scientific production [5, 13, 27, 28].

We identified nineteen subject clusters as sub-networks in the clinical teaching field. “Humanism”,“Clinical reasoning”,“Co-operative Behavior”,“Medical student education”, and”Tutorial strategies” were the five central themes in these subject clusters. This means that currently the main focus of clinical teaching has placed on these five themes. Yoo (2015), also investigated the newly appeared subjects of medical education in the articles published in one of the core journals of medical education that is “the Korean Journal of Medical Education (KJME)”. It did not use a comprehensive pool of data similar to our study that collected the data from the three main databases namely Scopus, Web of Science and Medline with thousands of related journals. Also, Yoo (2015) did not distinguish the medical education with clinical teaching or other irrelevant articles received by the (KJME). However, Yoo’s study also found that “professional behavior” and” medical humanities” along with some other themes, as the key subjects [8]. This shows that these two themes are the core in medical and clinical education. However, the present study offers a comprehensive image of knowledge map for medical and clinical education relying on its comprehensive data collection. Ji, YA(2018), also studied the “research topics and trends in medical education by social network analysis” and unlike our study found that “Computer-Assisted Instruction,” “Personal Staffing and Scheduling,” “User–Computer Interface,” “Professional Competency,” “Accreditation”,” Program Evaluation,” and “Educational Measurement,” appearing in the field of medical education, he concluded that in the years (2006–2015) medical technological devices have been extensively incorporated into medical practice [5]. Our findings supports the arguments made of Hartzband and Groopman (2009) in regard to turning to the principles of Oslerian medicine embracing the humanistic medicine [29]. It perhaps is the consequence of paying attention to patient centeredness, humanism and evidence- based practice as the major movements in medicine through the last decades [25]. Because, the basic principle of evidence-based medicine is about bringing the patients at the first and paying attention to the humanitarian part of practice by decreasing the medical errors. One of the limitations of visualization software created knowledge maps is that the map is not easily understood by non-experts. Therefore, it is not possible for readers to interact with the maps presented in the article. We know this as limitation of this study because readers will not be able to select a particular cluster or zoom on it. The other issue to mention is that the graphs are created by metadata collected from the research articles and graph is not responsible for the accuracy of the role of actors in the network. For example, if a node with highest centeredness, betweenness and closeness indicates that the institute, country or author is the most active actor in the network. But it does not deal with that if the authorship criteria declared by the ICMJE have been met in authorship. This may create ethical problems with the SNA. Also, similarity in name spelling may create problems in spiting the works of one author in various nodes and prevent creation of realistic score of the node. This may be easily solved by manual data curation before visualization, but the co-authorship features are not disclosed in metadata and remain unmasked in the network. We recommend content analysis and rigorous investigation of co-authorship from ethical point of view if the authorship network is the case.

## Conclusions

It is concluded that, a shift towards the consideration of humanity, humanism, moral and ethics in medicine is expected based on the high prevalence of concepts like “Humanism “in the current research outputs of the clinical teaching. In other word, higher volume of co-occurrence of concepts like “Humanism “and “Clinical reasoning “in the subject clusters addresses the need towards highlighting the cognitive dimension and human behaviors as of two important focus in clinical education. Density of clinical teaching network is weak. To improve cohesity of the authorship network of clinical teaching it is essential to improve research collaboration and co-authorship between new researchers and those who have better closeness or geodisk path with others, especially those with the clinical background. In this study top authors with closeness and betweennness have been introduced for authors and countries that may help with building collaboration ties internationally. It is recommended to analyse and visualize the research outputs of academic fields at list every 2 years, due to short half-life of the researches, to recognize the influencing authors and research centers who have potential for becoming the powerful leaders (node) of the knowledge network and to identify the new concepts to plan for future directions of research and education.

It is also suggested that medical universities regulate policies and provide opportunity for their researchers to team-work with educational and clinical specialists in other countries specially those who have powerful geodisk path in the network.

## Data Availability

Data are available through the authors upon reasonable request and upon the agreement of Iran University of Medical Sciences vice-deputy of Research.

## Abbreviations

SNA: Social Network Analysis
CC: Clustering Coefficient
PBL: Problem- Based Learning
MeSH: Medical Subject Headings
Ravar PreMap software: A free and native software for identifying co-occurrence of words and preparing data for Mapping, designed by Dr. Mohammad Tavakolizadeh, 2007 was used (http://mravari.blogfa.com)
VOSviewer software: Designed to construct and visualize bibliometric maps such as co-authorship, co-occurrence, and citation based maps by Van Eck and Waltman, 2009). VOSviewer software program, v.1.6.11, free and easy to use software for visualizing bibliometric network (https://www.vosviewer.com)

## Footnotes

1 Ravar PreMap software (a free and native software for identifying co-occurrence of words and preparing data for Mapping, designed by Dr. Mohammad Tavakolizadeh, 2007 was used (http://mravari.blogfa.com)

2 VOSviewer software (designed to construct and visualize bibliometric maps such as co-authorship, co-occurrence, and citation based maps by Van Eck and Waltman, 2009). VOSviewer software program, v.1.6.11, free and easy to use software for visualizing bibliometric network (https://www.vosviewer.com)

## References

[1] Beigzdeh A, Bahaadinbeigy K, Adibi P, Yamani N (2019). Identifying the challenges to good clinical rounds: a focus-group study of medical teachers. J Adv Med Educ Professionalism.

[2] Dent J, Harden RM, Hunt D (2017). A practical guide for medical teachers: Elsevier health sciences.

[3] Chang Y-H, Chang C-Y, Tseng Y-H (2010). Trends of science education research: an automatic content analysis. J Sci Educ Technol.

[4] Isba R, Woolf K, Hanneman R (2017). Social network analysis in medical education. Med Educ.

[5] Ji YA, Nam SJ, Kim HG, Lee J, Lee S-K (2018). Research topics and trends in medical education by social network analysis. BMC Med Educ.

[6] Saritas O, Burmaoglu S (2015). The evolution of the use of foresight methods: a scientometric analysis of global FTA research output. Scientometrics..

[7] Tabassum S, Pereira FS, Fernandes S, Gama J (2018). Social network analysis: an overview. Wiley Interdiscip Rev Data Mining Knowl Discov.

[8] Yoo HH, Shin S (2015). Trends of research articles in the Korean journal of medical education by social network analysis. Korean J Med Educ.

[9] Hazrati H, Gavgani VZ (2016). Which levels of education in medical sciences utilize most the Problem Based Learning?-A citation analysis study. Libr Philos Pract..

[10] Colacino C (2017). Medicine in a Changing World. [News and research].

[11] Zare-Farashbandi F, Geraei E, Siamaki S (2014). Study of co-authorship network of papers in the journal of research in medical sciences using social network analysis. J Res Med Sci.

[12] Fuhse JA (2015). Theorizing social networks: the relational sociology of and around Harrison white. Int Rev Sociol.

[13] Ghafouri HB, Mohammadhassanzadeh H, Shokraneh F, Vakilian M, Farahmand S (2014). Social network analysis of Iranian researchers on emergency medicine: a sociogram analysis. Emerg Med J.

[14] Vahed N, Gavgani VZ, Jafarzadeh R, Tusi Z, Erfanmanesh M (2018). Visualization of the scholarly output on evidence based librarianship: a social network analysis. Evid Based Libr Inf Pract..

[15] Bhattacharya S (2019). Some salient aspects of machine learning research: a Bibliometric analysis. J Sci Res.

[16] Bhattacharya S (2019). Some salient aspects of machine learning research: a bibliometric analysis. J Dent Sci Res..

[17] Bakkalbasi N, Krichel T (2006). Patterns of research collaboration in a digital library for economics. Proc Am Soc Inf Sci Technol.

[18] Gavgani VZ, Vahed N (2017). 184: Key Word Validity Ratio (KWVR); A Tool For Validity Of Keywords For Building An Accurate Literature Search Strategy. BMJ Open.

[19] Chang AA, Heskett KM, Davidson TM (2006). Searching the literature using medical subject headings versus text word with PubMed. Laryngoscope.

[20] Norman G (2011). Fifty years of medical education research: waves of migration. Med Educ.

[21] AlHaqwi AI, Taha WS (2015). Promoting excellence in teaching and learning in clinical education. J Taibah Univ Med Sci.

[22] Pradhan N, Gyanchandani M, Wadhvani R (2015). A Review on Text Similarity Technique used in IR and its Application. Int J Comput Appl..

[23] Muñoz-Leiva F, Viedma-del-Jesús MI, Sánchez-Fernández J, López-Herrera AG (2012). An application of co-word analysis and bibliometric maps for detecting the most highlighting themes in the consumer behaviour research from a longitudinal perspective. Qual Quant.

[24] Al-Qeicy AN, Ameen SY (2006). Network topology optimization using SocialNetwork analysis. J Eng Sustainable Dev.

[25] Borgatti SP (2006). Identifying sets of key players in a social network. Comput Math Organ Theory.

[26] Azer SA, Azer S (2019). Top-cited articles in medical professionalism: a bibliometric analysis versus altmetric scores. BMJ Open.

[27] Azer SA (2015). The top-cited articles in medical education: a bibliometric analysis. Acad Med.

[28] Rajabi F, Majdzadeh R, Ziaee SAM (2011). Trends in medical education, an example from a developing country. Arch Iranian Med (AIM)..

[29] Shelley BP (2014). Wither clinical skills and humanism?. Arch Med Health Sci.

